# Cell Death and Reproductive Regression in Female *Schistosoma mansoni*

**DOI:** 10.1371/journal.pntd.0001509

**Published:** 2012-02-21

**Authors:** Sarah E. Galanti, Stanley Ching-Cheng Huang, Edward J. Pearce

**Affiliations:** 1 Department of Pathobiology, School of Veterinary Medicine, University of Pennsylvania, Philadelphia, Pennsylvania, United States of America; 2 Trudeau Institute, Saranac Lake, New York, United States of America; 3 Department of Pathology and Immunology, Washington University School of Medicine, St. Louis, Missouri, United States of America; University of Queensland, Australia

## Abstract

The vitellarium is a highly proliferative organ, producing cells which are incorporated along with a fertilized ovum into the schistosome egg. Vitellarial growth fails to occur in virgin female schistosomes in single sex (female-only) infections, and involution of this tissue, which is accompanied by physical shrinkage of the entire worm, occurs when mature females sexually regress upon removal from their male partners. We have found that upon removal from their hosts into tissue culture, female parasites regress whether they are mated or not, but that cessation of egg production and a decline in expression of the vitelline gene p14 is delayed by mating. We used BrdU labeling to investigate whether there was a loss of proliferation in the vittelarium that might account for regression and found that the proliferation rate declined equally in paired and singled females once placed into culture. However, TUNEL staining and Caspase 3 activity measurements indicate that the loss of vitrellarial cellularity associated with regression is associated with profound apoptotic vitelline cell death, which is not apparent in the vitellaria of paired females immediately ex vivo, and which develops in vitro regardless of whether males are present or not. Furthermore, primordial vitellaria in virgin females have a high frequency of apoptotic cells but are characterized by a proliferation rate that is indistinguishable from that in fully developed vitellaria in mature paired females. Taken together, our data suggest that the vitelline proliferation rate is independent of pairing status. In contrast, the survival of vitelline cells, and therefore the development of the vitellarium, is highly male-dependent. Both processes are negatively affected by removal from the host regardless of whether male worms are present or not, and are unsustainable using standard tissue culture approaches.

## Author Summary

Schistosomes are parasitic trematode worms that infect more that 200 million people in 76 countries of the tropics and subtropics. These parasites are unusual amongst trematodes in having separate sexes. Mating of male and female schistosome involves the female residing within a specialized canal on the ventral surface of the male. Full sexual maturation of the female is dependent upon her residence within this niche. Sexual maturation involves the development of the vitellarium, a tissue that contributes critical cells to the egg. Remarkably, the vitellarium never grows in virgin females and regresses in mated female parasites once they are removed from males. Our study aimed to understand the basis for vitellarial growth and regression. We have found that the vitelline cells within the organ proliferate independently of males but are dependent on male parasites for their survival. Both cellular proliferation and death within this organ are negatively affected by removal from the host regardless of whether male worms are present or not, suggesting the presence within the host of a key factor that is not represented in standard tissue culture medium.

## Introduction

Infection with trematode parasites of the genus *Schistosoma* causes chronic and debilitating disease in over 200 million people worldwide [1], [2]. Adult *S. mansoni* worms live within the mesenteric veins laying eggs that are intended to pass into the intestinal lumen for release into the environment to continue the life cycle and allow transmission of the infection [3]. However, because blood within the portal vasculature flows away from the intestine, many eggs are carried to the liver, where they become trapped in sinusoids, and elicit strong Th2 cell mediated immunopathology which is the cause of disease manifestations [3]. Since egg production is key for both transmission and pathogenesis, studying the mechanisms involved in schistosome reproductive development could lead to new methods of preventing or treating disease [4].

Unique among parasitic trematodes, adult schistosomes exhibit sexual dimorphism and display an interesting codependency: the female resides in a groove, the gynecophoric canal, on the ventral side of the male and ongoing physical pairing (but not sperm transfer [5]) is necessary for proper sexual development [6]–[12]. Virgin female schistosomes, from female-only infections, are developmentally stunted compared to females from mixed-sex infections, exhibit underdeveloped vitellaria and ovaries, and are unable to lay eggs [12], [13]. Furthermore, egg-laying females that are physically separated from their male partners and are surgically implanted into a host in the absence of male worms cease egg laying and regress reproductively to an immature state. Interestingly, this regression is reversible because normal reproductive activity is resumed when separated females are re-paired with males [12], [14], [15].

Much of the change in overall size of a female worm as it sexually matures or regresses is due to changes in the vitellarium. The vitellarium is a proliferative tissue that occupies the posterior two thirds of the female and produces cells that surround the ovum and provide the precursor proteins for eggshell formation and nutrients for the developing embryo. It contains cells (vitellocytes) in 4 morphologically distinct stages of development [16], [17], with the most mature stage-4 cells being characterized by electron dense vitelline droplets that contain eggshell precursor proteins such as p14, p19 and p48 [18], [19]. The vitellaria of virgin females, as compared to mature paired females, contain only stage-1 vitellocytes [17]. Paired females have also been reported to have more systemic mitotic activity than virgin females as shown by incorporation of tritiated thymidine, with the most densely labeled cells being stage-1 vitellocytes [20]. Moreover, transcription of a number of genes, including p14, within vitellocytes has been reported to be dependent on pairing [21], [22]. Upon separation from males, there is a reported decrease in uptake of tritiated thymidine by the female, and cessation of expression of genes that are specifically expressed in vitelline cells, that are reversed upon repairing [20] [22]. Based on these previous findings, it is reasonable to assume that the proliferation of stage-1 vitellocytes and subsequent differentiation of daughter cells into stage-4 cells is the process that accounts for the growth of the vitellaria, and therefore of the entire worm, in sexually mature female worms.

A hallmark of long-lived metazoans is their ability to regenerate adult tissues. This process is exemplified by the development of the mammary gland due to the proliferation of mammary epithelial cells (MEC) in response to reproductive hormones, and the involution of the same tissue due to widespread MEC apoptosis following the cessation of lactation [23]. Cell death due to apoptosis has also been implicated in tissue remodeling prior to regeneration in free-living platyhelminths. These findings raise the intriguing possibility that vitellarial development and involution is regulated at some level by a balance between cellular proliferation and programmed apoptotic cell death. Availability of the genomic sequence of *Schistosoma* revealed that, as would be expected, key genes necessary for apoptosis are encoded in these worms [24], and recent reports have revealed that the apoptotic pathway is active in schistosomes [25]. Here we address the relative contributions of failings in cellular proliferation or increased apoptotic cell death in the regulation of vitellarial cellularity. Our findings indicate that the absence of male worms leads to profound increases in vitelline cell apoptosis and that this, rather than decreased proliferation within the vitellarium is likely to underlie developmental failures in this tissue in the absence of male parasites.

## Materials and Methods

### Ethics statement

All experimental use of animals for the studies described in this paper were performed in accordance with the recommendations of the U.S. Government Principles for the Utilization and Care of Vertebrate Animals Used in Testing, Research, and Training and approved by the Institutional Animal Care and Use Committees of the University of Pennsylvania and the Trudeau Institute. Mice infected with schistosomes for the experiments described herein did not exhibit symptoms of disease associated with infection.

### Parasites and animals

The Puerto Rican/NMRI strain of *S. mansoni* was used in all experiments. Adult schistosomes were recovered by hepatic-portal perfusion from C57BL/6 female mice (The Jackson Laboratory) that had each been percutaneously exposed to ∼150 cercariae 7 weeks earlier. Adult parasites were maintained in vitro in M199 (Gibco), 10% fetal calf serum, 2% Antibiotic/Antimycotic (Gibco), and 1% HEPES, supplemented with whole blood from an uninfected mouse (0.5%) in a 37°C/5% CO_2_ atmosphere. Parasites were cultured at 5 pairs or 10 females in 4 ml medium per well of a 6-well plate. The media was changed every 48 h. Adult pairs were separated manually by gentle stroking with a fine hair-loop tool. To specifically measure egg production, 4 females were cultured, paired or unpaired, per well of a 6-well plate. After each 24 h period, the worms were transferred to fresh wells and eggs remaining within the well were counted using a gridded dish and a dissecting microscope. For parasite measurements, females were photographed in culture dishes using a Leica DC500 camera attached to a Leica MZ6 stereoscope, and the cross sectional surface area was calculated by tracing the worms in Openlab 3.5.1 (Improvision).

### Real time RT-PCR

Worms were collected at designated time points and frozen in RNAlater (Qiagen) until RNA extraction. Total RNA was extracted from parasites using RNeasy (Qiagen), and contaminating genomic DNA was removed by DNase treatment using Turbo DNA-free endonuclease (Ambion). First-strand cDNA was synthesized using equal amounts of RNA, SuperScript II reverse transcriptase (Invitrogen), and oligo dT as a primer. RT-minus controls were performed to confirm absence of genomic DNA (data not shown). P14 and P19 transcript levels were quantified relative to α-tubulin using Applied Biosystems' 7500 real-time PCR system and SYBR green PCR Master Mix (Applied Biosystems), and the 2^−ΔΔCt^ method. Dissociation curves were generated for each real-time RT-PCR to verify the amplification of only one product.

P19 primers were: forward 5′-TGCTGCATATGGAAGTGGTT-3′ and reverse 5′-TCATTTGATGATTCTCCATTGTTT-3′. P14 primers were: forward 5′-ACAGTCACTCACACTCGTCTTCTT-3′ and reverse 5′-GCCATAACCGCTATCACAATC-3′. α-Tubulin primers were: forward 5′-TAGAGCGTCCAACCTACACAA-3′ and reverse 5′-GGAAGTGGATACGAGGATAAGG-3′.

### DNA quantification

Relative cell numbers were inferred by measuring total DNA per 5 females. DNA was quantified using Quant-iT (Invitrogen). Briefly, worms were ground in lysis buffer (140 mM NaCl, 50 mM Tris-HCl pH 7.4, 0.1% Triton-X100, and a protease inhibitor cocktail (Roche Diagnostics, Mannheim, Germany), and fluorescent dsDNA dye was added. Fluorescence was quantified on a fluorometer as per the manufacturer's instructions.

### Tissue sections

Parasites were fixed in 4% paraformaldehyde for 1–2 hours at room temperature, dehydrated, and embedded in paraffin. All staining was performed on slides containing 5 µm sections of 4–6 females.

### TUNEL staining

Sections were deparaffinized in xylene, rehydrated, washed with PBS and treated with 20 µg/ml of Proteinase K (Roche) for 10 min at room temperature. Terminal deoxynucleotidyl transferase nick end labeling (TUNEL) reactions were conducted using Apoptag (Chemicon) according to the manufacturer's instructions, and detected using Cy3-anti-digoxygenin (Jackson). Microscopy was done on a Nikon E-600 microscope equipped with a QICam Fast 1394 camera (Q Imaging) and IVision imaging software (BioVision Technologies).

### Caspase 3 activity

The *S. mansoni* genome encodes a caspase 3 like gene and previous reports have measured caspase 3 activity in schistosomes [26]. Parasites were collected and washed once with 0.5 ml of sterile water. Worms were then immediately homogenized in 1× lysis buffer (Caspase 3 assay kit, Sigma) at a concentration of 6 worms per 100 µl of buffer; incubated and centrifuged (20,000× *g*) at 4°C for 15 min and 20 min respectively. Five microliters of the supernatant were used per assay. Caspase-3 activity was determined through the absorbance of p-nitroanilide (pNA) cleaved from the substrate acetyl-Asp-Glu-Val-Asp p-nitroanilide (Ac-DEVD-pNA) at 405 nm, and normalized by total protein concentration.

### BrdU incorporation and staining

Parasites were labeled *in vitro* by adding BrdU to the culture media at a concentration of 1 mM for the designated period of time. Parasites were labeled *in vivo* by intraperitoneal injection of infected mice with 10 mg BrdU. Parasites were fixed and sectioned as above. Sections were deparafinized and rehydrated as above. Sections were microwaved in citrate buffer, pH 6.0, to denature dsDNA to expose the BrdU. Slides were blocked with Superblock (Fisher) and incubated overnight at 4°C with rat anti-BrdU 1∶1000 (Accurate Chemical and Scientific Corp.), then detected using Cy3-anti-rat 1∶600 (Jackson). Microscopy was done on a Zeiss Axiovert 200 M using AxioVision 4.6 software.

### Statistical analyses

When possible, data were analyzed statistically. Details of statistical analyses are presented in Figure Legends.

### Accession numbers

Accession numbers of genes and sequences used in this study are available from GenBank (http://www.ncbi.nlm.nih.gov/Genbank) and include the following: *S. mansoni* P14 (GI: 10176); *S. mansoni* P19 (GI: 418114); *S. mansoni* caspase-3 (GI: 256576819); *S. mansoni* alpha tubulin-1 (GI: 8355916).

## Results

### Paired and separated females shrink and lose vitellarial mass when placed into tissue culture

**Figure 1:**
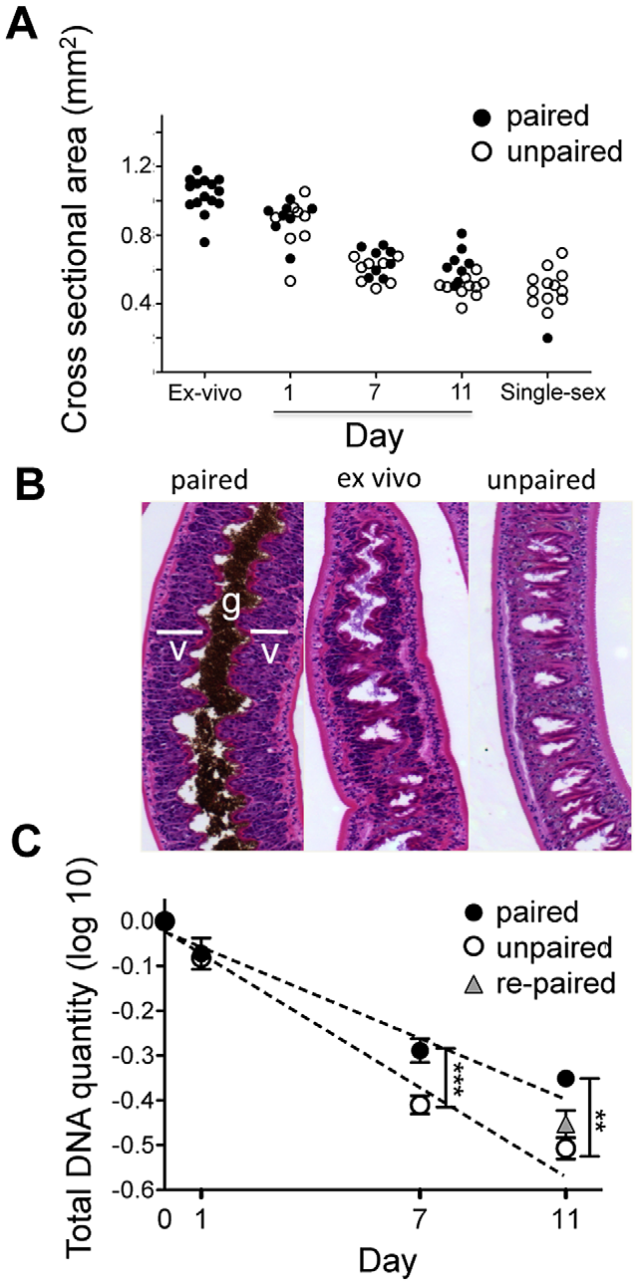
Paired and separated females shrink and lose vitellarial mass when placed into tissue culture. Adult schistosomes were placed in culture paired with male worms, or as singled females, and followed over time. A) Changes in cross sectional area of females over time in culture, and compared to freshly recovered paired females (ex-vivo) and virgin females from single sex infections. The slope of decline in size is slightly steeper for un-paired females; although the difference is statistically significant, it is so small that it is unlikely to be biologically significant. Data were log transformed for analysis, but not display. Linear regression analysis revealed a significant (P<.005) difference in the slopes, but there was no significant difference between the size of paired and un-paired females at any time point when compared independently using t-tests. Each data point represents a single female worm. B) H&E stained sections of a freshly recovered female parasite (Ex-vivo) and females that had been maintained as paired or singled worms in vitro for 7 days. The location of the vitellarial tissue (v) and gut (g) in the paired ex-vivo female are shown for reference. C) Total DNA was measured relative to a standard curve with known quantities of DNA, and normalized to the ex vivo control (day 0). Data was log transformed for analysis. Linear regression analysis revealed significant (P<0.01) difference in the slopes of DNA loss between paired and singled (un-paired) females, and t-test showed significant difference between paired and un-paired females at day 7 (P<0.001) and day 11 (P<0.01). Day 11 DNA levels in singled females that were re-paired with males at day 7 were no different from those in females that remained singled through this time.

We analyzed the relative effects on overall size and on vitellarial cellularity, of removing schistosomes from infected mice and placing them in culture paired with the male worms that they were recovered with, or as unpaired females separated from males. We noted that rapidly over time female parasites (but not males, data not shown) began to shrink and that the rate of shrinkage was equivalent in the presence or absence of males. After 11 days in culture, paired or separated female schistosomes were the same size as virgin females recovered from single sex infections (Fig. 1A). Loss of mass was accompanied by clear loss of cellularity of the vitellaria (Fig. 1B), and a striking, accompanying decline in overall DNA content that was consistent with the apparent loss of cells from the worms (Fig. 1C); a reduction in DNA content was not detected in cultured male parasites (data not shown). Interestingly, vitellarial cellularity was maintained to a greater degree in paired females than in singled females (Fig. 1B), and this was reflected in a slower rate of decline in DNA content in the paired vs. singled worms (Fig. 1C). Addition of male worms to separated females at day 7 of culture failed to significantly reduce the decline in DNA loss (Fig. 1C).

### Paired and unpaired females stop laying eggs and expressing genes encoding egg protein when placed into tissue culture, but the rate of decline is more rapid in the absence of male schistosomes

**Figure 2:**
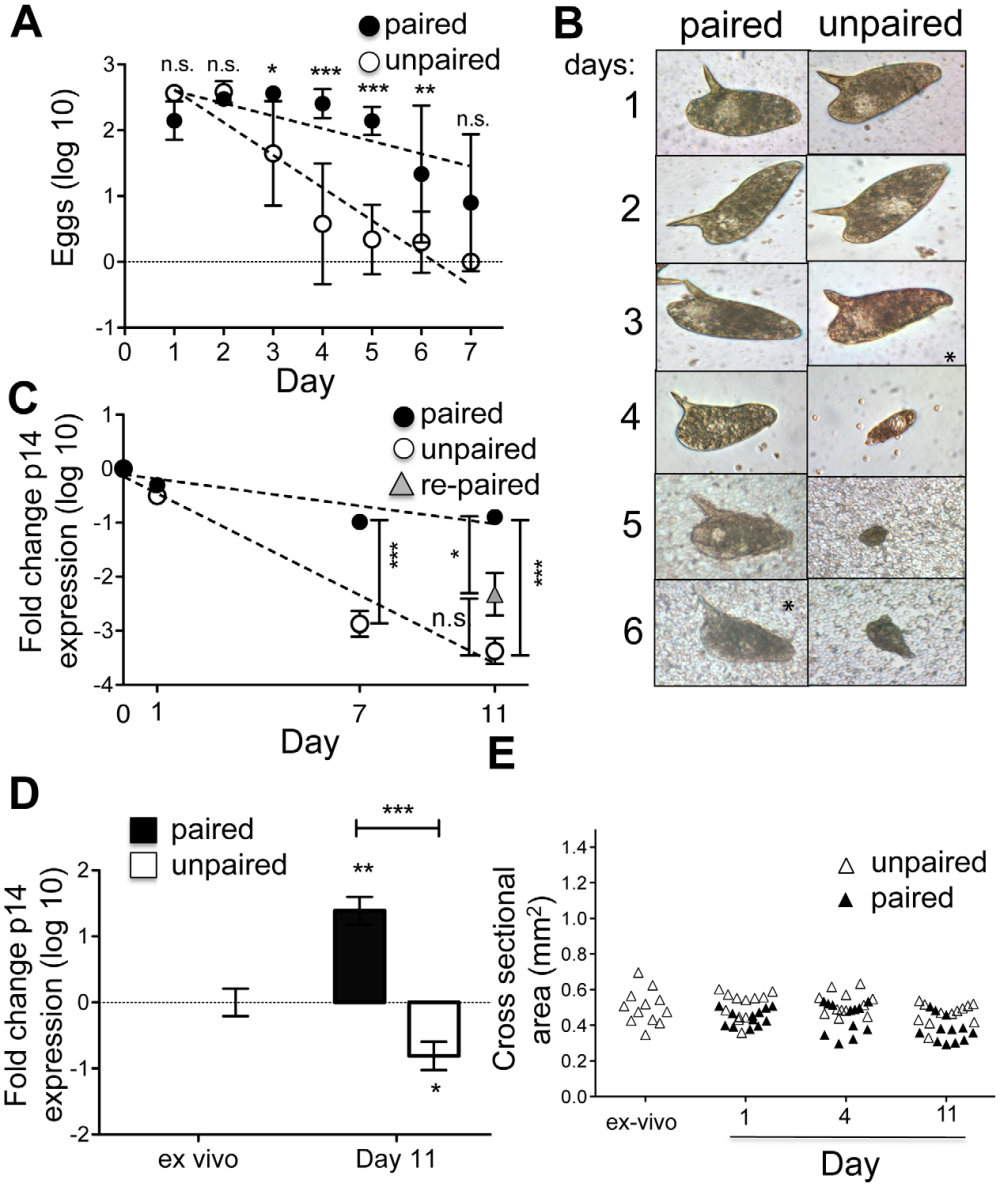
Females stop laying eggs and expressing genes encoding egg protein when placed into tissue culture. Adult females from mixed sex infections were placed in culture with or without male parasites (Four female parasites per well of a 6 well plate). A) Total numbers of eggs per well produced during each 24 h period were counted. Results are pooled from 3 individual experiments. The rate of decline of egg production is significantly faster in un-paired females. Data were log transformed (Y = log(Y+1)) for analysis. Linear regression analysis revealed a highly significant (P<0.0001) difference in the slopes of egg production decline. A 2-way-ANOVA showed a significant interaction of pairing and time on egg production, and Bonferroni post-test determined significant effects of pairing on egg production at days 3 through 6. B) Eggs produced during each day of culture were photographed. C) Real-time RT-PCR analyses of p14 transcripts. Expression of S. mansoni alpha-tubulin was used as the endogenous control to calculate relative expression levels, and expression levels were normalized to ex vivo females. Data were log transformed for analysis. Expression of P14 declined significantly over time in culture in both paired and un-paired females. Linear regression analysis revealed a highly significant (P<0.0001) difference in the slopes of p14 expression decline between paired and un-paired females. Independent t-tests performed at each time point showed dramatic and highly significant differences in expression of p14 at days 7 and 11 between paired and un-paired females (P<0.0001). Females that were un-paired for 7 days in vitro, then re-paired for 4 days showed a trend of higher P14 expression at day 11 than females who were un-paired for the full 11 days. This suggests that re-pairing with the male may be sufficient to prevent further decline of eggshell protein expression. There were no significant changes in alpha-tubulin expression over the time period of the experiment. D) Real time RT-PCR analyis of p14 transcripts in virgin females from single sex infections following culture in vitro paired or un-paired with male parasites. Only worms that were actually paired for the 11day duration were considered paired. Expression of alpha-tubulin was used as the endogenous control to calculate relative expression levels, and expression levels were normalized to the ex vivo females. Expression of P14 increased significantly (P<0.01) in single sex females that were paired for 11 days in vitro. Furthermore, the already low expression of P14 in virgine females from single sex infections was further reduced upon culturing in the absence of males (P<0.05). Thus, the difference in P14 expression levels at day 11 in females that are paired vs un-paired is highly significant (P<0.0001). Data were log transformed for analysis. T-tests were done to compare expression between samples. E) Changes in cross sectional area of virgin females from single sex infections over time in culture with or without males. No significant differences in size over time with or without males were noted. Each data point represents a single female worm.

We measured egg production by both paired and separated female schistosomes ex-vivo. Unpaired separated females exhibited a rapid and pronounced decline in egg production that was not apparent in the cultures of paired females (Fig. 2A). Nevertheless, over time, paired female parasites also stopped producing eggs (Fig. 2A). The morphology of eggs produced in culture was normal for both paired and unpaired females until production levels began to decline, after which newly formed eggs were small and aberrantly shaped (Fig. 2B). These changes in egg production were mirrored by decreases in expression of the gene encoding the egg protein p14, which continued to be expressed in vitro in paired females for longer than in singled worms, but nevertheless declined in both over time (Fig. 2C). We attempted to reverse the decline in p14 expression by repairing separated un-paired females with freshly isolated ex-vivo males for the last 4 days of an 11 day culture. This process was partially successful in that expression of p14 was higher in re-paired worms than in separated females, although it remained below that evident in females that had been paired for the entire 11 day period of culture (Fig. 2C). The ability of male worms to stimulate p14 expression was emphasized by an observed 30-fold increase in p14 expression in virgin females from single sex infections that were cultured with male parasites for 11 days (Fig. 2D). However, overall levels of p14 expression in this situation remained lower than in ex-vivo worms (data not shown), and increased p14 expression was not accompanied by worm growth (Fig. 2E).

### Involution of the vitellarium is associated with significant apoptosis within the vitelline cell population

**Figure 3:**
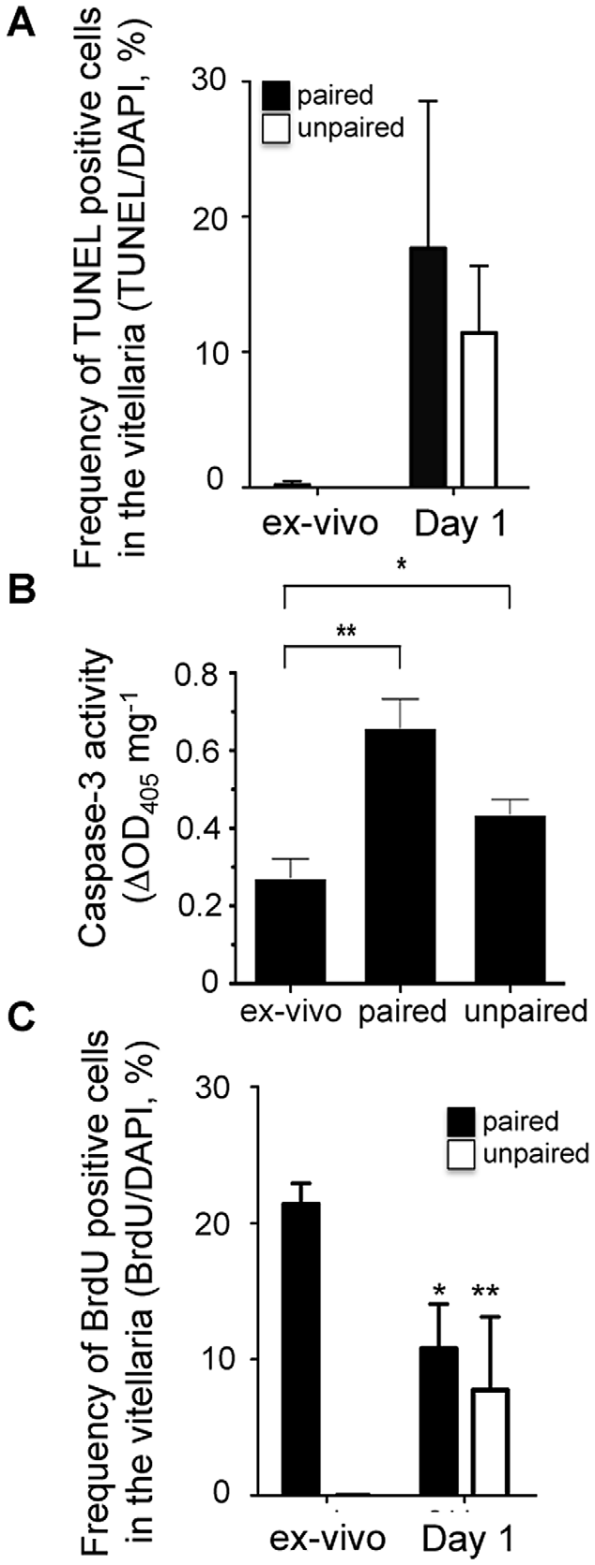
Involution of the vitellarium is associated with significant apoptosis within the vitelline cell population. Adult females were recovered from mixed sex infections and placed in culture as paired or singled, un-paired worms for 1 h in the presence of BrdU, or cultured for 24 h, with BrdU added at the 23^rd^ hour. Worms were fixed, sectioned and stained with TUNEL and DAPI (A) or with anti-BrdU and DAPI (C). The ratios of double positive cells to DAPI single positive cells within the vitellaria were enumerated microscopically. TUNEL-positive cells were extremely rare in worms examined within 1 h of recovery. However, all females examined exhibited a significant increase in apoptotic vitelline cell death within 24 h (A). In contrast we measured a significant reduction in the frequency of proliferating cells over this same time period (C) (p<0.01 in paired and in singled worms, as assessed by Student's t-test). B) Casapse-3 activity was monitored from the cell lysates of ex-vivo, paired and unpaired female worms. Results are given as an optical density of 405 nm normalized by parasite total protein concentration (**, *p*<0.01; *, *p*<0.05, as assessed by Student's t-test).

We reasoned that vitellarial involution in females in tissue culture could be due either to a failure of cells within this organ to proliferate, or to increased cell death. To examine this we labeled worms in vitro with BrdU and then used anti-BrdU antibodies in conjunction with DAPI and TUNEL-staining on sections of female parasites to identify proliferating and apoptotic cells respectively. We noted a significant increase in the percentage of TUNEL-positive cells in the vitellelaria of both paired and unpaired female parasites within 24 h of culture (Fig. 3A). This was accompanied by a significant increase in caspase 3 activity in these worms, supporting the view that apoptosis rapidly increases in females parasite once removed from the host (Fig. 3B. In light of the fact that vitellarial decline is delayed by pairing, it is unclear why caspase 3 activity was higher in paired females compared to unpaired females in these analyses). The frequency of TUNEL positive cells had declined by day 7, indication that most cell death occurred rapidly following removal from the host (Table 1). Of note, we did not observe TUNEL-positive nuclei in intestinal or other somatic tissues, suggesting that cell death associated with removal of parasites from the host is primarily occurring within the reproductive organs (Table 1). Within the same 24 h period we also noted that the proliferation index declined significantly, regardless of whether females were paired or singled (Fig. 3C). Both increased apoptosis and decreased BrdU incorporation occurred within paired and unpaired separated females, leading us to conclude that vitellarial involution in vitro results from a combination of increased apoptosis and decreased proliferation that is not prevented by the presence of male parasites.

**Table 1:** Frequency of TUNEL positive cells in specific tissues of female *S. mansoni*.

	vitellaria	intestine	other tissues
**ex vivo**	0%	0%	0%
**24 hr paired**	15%	0%	0%
**24 hr singled**	13%	0%	1%
**7 d paired**	3%	0%	0%
**7 d singled**	9%	0%	0%

**Figure 4:**
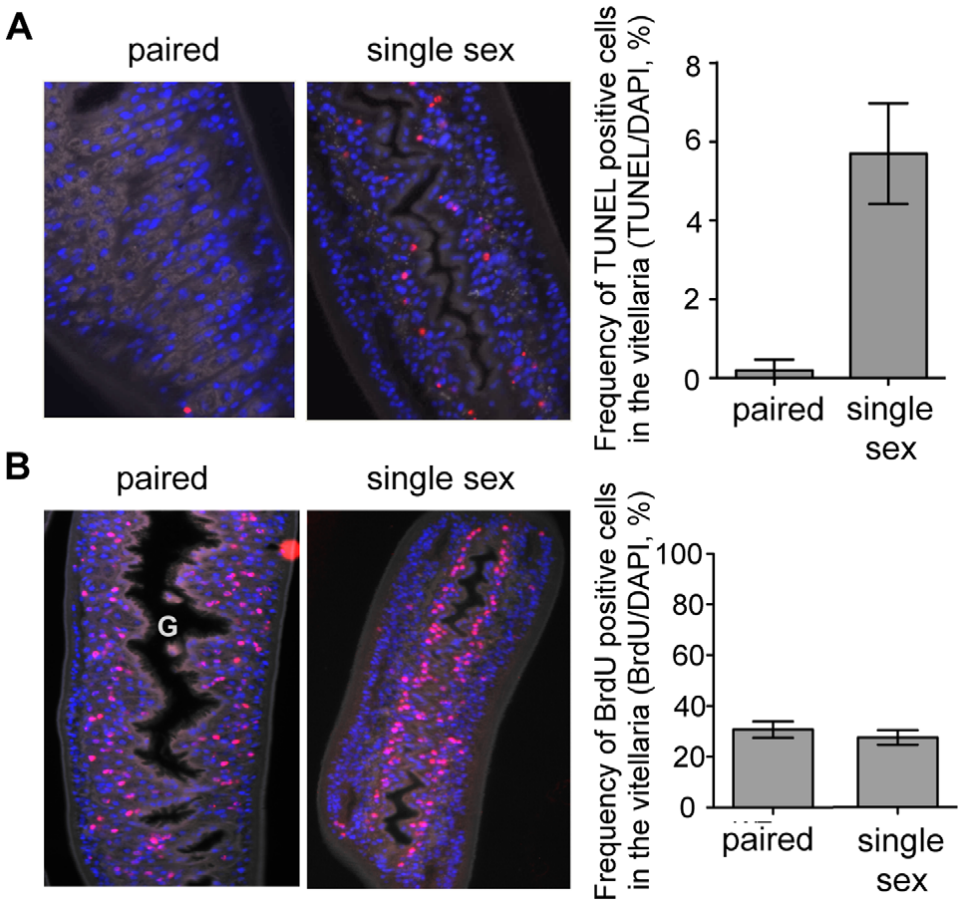
Viteline cells are proliferating in paired females and in unpaired females from single sex infections. In contrast, apoptosis is occurring only in the vitellarial tissues of single females. Mice infected with mixed sex or female-only parasites were injected with BrdU 24 h prior to sacrifice. Recovered parasites were fixed, sectioned and stained with TUNEL and DAPI (A) or with anti-BrdU and DAPI (B). A) TUNEL positive cells were more frequent in the vitellaria of females from single sex infections (P<0.0001) compared to ex vivo females from a mixed sex infection. Data was log transformed for analysis. Significance was determined by t-tests assuming unequal variances. TUNEL positive cells occurred almost exclusively in the vitellaria (data not shown). DAPI (blue), TUNEL (Red). Autofluorescence in the FITC channel is shown in grey for tissue context. 40× magnification. G = gut. B) The frequency of BrdU positive cells in the vitellaria of females from mixed sex infections and single sex infections were not significantly different. DAPI (blue), BrdU (red). Autofluorescence in the FITC channel is shown in grey for tissue context. 20× magnification.

Lastly, we examined whether there were differences in cellular proliferation or apoptosis in the virgin females from female-only infections vs. paired females from mixed sex infections. For these experiments we labeled worms in vivo with BrdU, then recovered them from their infected hosts and immediately fixed them in preparation for sectioning and staining with anti-BrdU antibodies, TUNEL and DAPI. We found a dramatically increased frequency of TUNEL staining in females from single sex infection vs. mixed sex infections, and apoptotic cells were almost entirely restricted to the vitellaria (Fig. 4A). In contrast, the frequency of BrdU labeled cells in the vitellaria was the same in females from mixed sex and female-only infections (Fig. 4B). In females isolated from a mixed-sex infection and paired with a male parasite, BrdU-incorporating cells seemed to be randomly distributed throughout the vitellaria. However, in virgin females the proliferating cells were tightly aligned along the intestine within the undeveloped vitellarial primordium. These data suggest that a lack of development of the vitellarial tissues without male parasites is due primarily to the death of cells that are being produced through a proliferative process that is male-independent.

## Discussion

Here we set out to analyze the underlying mechanism for the growth and regression of the vitellarial tissues in female schistosomes. There is clear documentation that virgin female schistosomes remain stunted and fail to sexually mature in the absence of male parasites, and that mated females sexually regress and physically shrink when removed from male schistosomes. To a large extent, development and regression of female worms reflect changes in the activity and cellularity of the vitellaria. We hypothesized that loss of vitellarial cellularity could be due to the death of vitelline cells, the cessation of proliferation of cells within this organ, or a combination of both of these events. Moreover, these events would be expected to be strongly influenced by the presence of male parasites. Our findings suggest that in vivo, failure of vitellarial development in the absence of male parasites is largely the result of ongoing apoptosis, and not due to a lack of cellular proliferation within this organ. Moreover, while clearly sufficient to allow and sustain female development in vivo, male parasites are insufficient to prevent vitelline cell apoptosis, normal vitelline cell proliferation, vitellarial atrophy, or female sexual regression in vitro, suggesting that an additional factor(s) present in the host, but absent in our tissue culture conditions, is playing a critical role in female reproductive tract health.

Previous reports using scintillation counting or dot-blotting to detect incorporation of ^3^H-thymidine or of the thymidine analog BrdU, respectively, as measures of cellular proliferation, concluded that changes in the cellularity of vitellaria were largely the result of changes in proliferation, and that male parasites provide a positive signal for vitelline cell proliferation [20], [27]. The findings went further to identify stage 1 vitellocytes as the most densely labeled of 4 identified types of vitellocyte [20]. These findings suggested a model in which vitelline cells proliferate due to the presence of male worms, and that vitellarial involution is presumably the net result of the cessation of proliferation and the packaging of existing vitelline cells into eggs during the first few days in vitro when female parasites continue to produce eggs. This is feasible since each egg requires approximately 40 vitelline cells and females can lay 200–300 eggs each day [12]. Nevertheless, our data do not entirely fit this model. Rather, by using microscopy to count cells that had incorporated BrdU, we found that: 1) the proliferation rate within the vitellaria of virgin females in vivo is similar to that in paired females in vivo; 2) the proliferative rate of vitelline cells in females decreases similarly within 24 h of being placed in culture as separated worms or as paired worms. We interpret our data as indicating that cell proliferation within the vitellarium is homeostatic and male-independent, but dependent on factor(s) present in the host and absent in tissue culture.

Our data indicate that the major event that is responsible for vitellarial failure in female parasites is vitelline cell death by apoptosis. Despite the fact that key genes of the apoptotic machinery are present in the schisosome genome, proof of the presence of active apoptotic pathways in schistosomes has been reported only recently [25], [26], [28]. Our report indicates that apoptosis is rare in healthy mated male and female schistosomes, but is ongoing within the vitellarial tissues of virgin female parasites in single sex infections. This finding indicates that male parasites supply a signal that is required for the survival of vitelline cells. Ex vivo, vitelline apoptosis occurs rapidly whether female parasites are mated or separated from their male partners, indicating that a key factor available in vivo is also either directly required for vitelline cell survival, or required for male parasites to exert their positive effects on the vitellaria. At this time the fate of apoptotic vitelline cell bodies is unknown. In other metazoans, apoptotic cell bodies are generally cleared by phagocytic cells [29], but whether phagocytes exist in schistosomes is unclear.

Our findings on the development and regression of schistosome vitellaria are consistent with the understanding of tissue remodeling developing from studies of planarians, free-living platyhelminths. In these organisms, which are amenable to more detailed analysis than are schistosomes, physical tissue damage or prolonged starvation leads to increased rates of apoptosis, which contribute to the restoration of anatomical form and function [30], [31]. Regeneration in this system is mediated by adult stem cells which proliferatively produce daughter cells that differentiate into specific cell types as required [30]. It is tempting to extrapolate from the planaria system, and our findings and previous reports on schistosome vitellocytes [20], to hypothesize that the ability of the vitellarium to develop and regress over numerous cycles is due to the presence within this organ of long-lived proliferative stem cells (perhaps stage 1 vitellocytes). We are investigating this possibility.

While our findings reveal that the failure of vitellarial development, or the involution of developed vitellarial tissues, is due to increased apoptotic cell death, they fail to identify the factor(s) that are responsible for maintaining vitelline cellularity. However, there have been numerous suggestions that male parasites promote female maturation by “providing” key nutrients (e.g. [32]). The fact that starvation in planaria can lead to reversible tissue involution through apoptosis is consistent with the possibility that vitelline cell loss is the end result of nutritional deprivation in female parasites [31]. Schistosomes are dependent on an environmental source of fatty acids and sterols since they are incapable of synthesizing these directly [24], and reported observations that males are able to transfer cholesterol and metabolites of cholesterol to females [33] [34] suggest that male parasites may play a crucial role in acquiring these molecules for females. This is interesting in light of the identification of a steroid hormone receptor, retinoid X receptor homologue, that binds to regulatory elements of the p14 gene [35]. It is plausible that female schistosomes need male-derived cholesterol in order to make steroid hormones that are essential for vitelline cell differentiation and the accompanying expression of key genes, such as p14, involved in egg production [35]. The idea that vitellarial regression, or the failure of this tissue to develop in the first place, is effectively the result of starvation, is compatible with the observed effects of tissue culture on paired and unpaired females, since it is conceivable that regardless of the presence of male parasites, culture conditions are failing to provide key nutrients that would normally be available in vivo. Moreover, it is reported that when a more complex culture medium than that used in the present study is utilized [36], [37], male parasites can to some extent promote the development of vitellarial tissues in virgin females in vitro, so it is feasible that to a greater or lesser extent, nutrient availability coordinated by males is the key event controlling vitelline cell survival in female parasites.

Based on our work, and that of others, it seems likely that growth factors in the TGFβ signaling pathway, and that activate cytoplasmic tyrosine kinases of the Src class [38], are involved in vitelline cell proliferation, differentiation, gene expression and subsequent egg production in schistosomes [18], [38], [39]. Of importance for the discussion here, a key role of growth factors is to ensure that cells are able to access available essential nutrients. For example, interleukin 2, the key growth factor for T lymphocytes in the mammalian immune system, promotes surface expression of a glucose transporter that allows proliferating T cells adequate access to extracellular glucose which fuels the glycolytic metabolism that underpins their proliferative program [40]. Whether males are simply providing nutrients directly to females, or allowing females to use nutrients by providing them with essential growth factors, is unclear at present. An interaction between a growth factor and its specific receptor may be compatible with the observation that schistosome males are better able to stimulate egg production by females of the same species compared to different species within the same genus [41]. If the putative growth factor is expressed on, or secreted from, the tegumental surface within the gynecophoric canal, it may also explain why sections of male worms (which, like whole worms, are able to clasp females within the gynecophoric canal) can stimulate vitellarial development within the part of the female that is within the canal, but not elsewhere in the same worm [37]. Localization of the putative signal to the gynecophoric canal might also provide a partial explanation for the early observation that virgin males brought into the gynecophoric canals of mature males eventually begin to develop and express hermaphroditic characteristics and make vitelline-like cells [6].

We hypothesize that in addition to directly supplying nutrients, male parasites provide a growth factor signal that allows female parasites to access key nutrients, and that this process is of value only if the nutrients are present in the environment. Supportive of the idea that female parasites are receiving signals and/or nutrients from male parasites in vitro is our finding that males can induce p14 expression in females in culture, a result that is compatible with previous reports that extracts of males worms are able to promote the development of vitelline cells [42] and the expression of p14 in female parasites [43]. Of note however, in our hands p14 expression is not sustained, nor indicative of wholesale vitelline cell survival in this setting, indicating that other key requirements are missing. We are actively pursuing this area in ongoing studies.
